# The limited reach of surprise: Evidence against effects of surprise on memory for preceding elements of an event

**DOI:** 10.3758/s13423-021-01954-5

**Published:** 2021-06-25

**Authors:** Aya Ben-Yakov, Verity Smith, Richard Henson

**Affiliations:** MRC Cognition and Brain Sciences Unit, University of Cambridge, Cambridge, UK

## Abstract

When reflecting on the past, some of our strongest memories are for experiences that took us by surprise. Extensive research has backed this intuition that we are more likely to remember surprising moments than mundane ones. But what about the moments leading up to the surprise? Are we more likely to remember those as well? While surprise is a well-established modulator of memory, it is unknown whether memory for the entire event will be enhanced, or only for the surprising occurrence itself. We developed a novel paradigm utilising stop-motion films, depicting of a sequence of narrative events, in which specific occurrences could be replaced with surprising ones, while keeping the rest of the film unaltered. Using this design, we tested whether surprise exerts retroactive effects on memory, and specifically whether any potential effect would be confined to elements in the same event as the surprising occurrence. In a large cohort of participants (*n* = 340), we found strong evidence that surprise did not retroactively modulate memory, neither when participants were tested immediately after study nor when they were tested 24 hours later. We suggest two possible accounts for these findings: (1) that the components of an event are encoded as independent episodic elements (not as a cohesive unit), or (2) that surprise segments experience, sectioning off the preceding elements as a separate event.

**Supplementary Information:**

The online version contains supplementary material available at 10.3758/s13423-021-01954-5.

You’re having dinner at a restaurant with friends, and just as you finish the starters, there’s a sudden blackout, so you continue your dinner to the light of cell phones and candles. That is certainly an evening you will remember. But will you remember only the blackout itself, or also what you had as a starter, before the blackout occurred? Extensive research has demonstrated that we are more likely to remember surprising occurrences, which mismatch our expectations (Ranganath & Rainer, 2003; von Restorff, 1933). Yet it is unknown whether surprise affects memory for other elements within the episode in which it occurred.

One consequence of surprise may be increased arousal. Various theoretical accounts converge into two opposing hypotheses regarding the potential penumbra effects of arousing stimuli. While several of these focus on a particular type of arousal (e.g., due to emotion or stress), the hypotheses put forward may be applicable to arousal in general. An object-based framework (Mather, 2007) merges the priority-binding theory of emotional stimuli, the cue-utilization hypothesis of emotion (Easterbrook, 1959), and a fragmentation account of traumatic memory (Payne et al., 2004). According to this framework, arousal leads to focused attention on the arousing object, impairing encoding and consolidation of surrounding information, including information held in working memory. This hypothesis is supported by findings that both aversive stimuli (Bornstein et al., 1998; Hurlemann et al., 2005; Knight & Mather, 2009; Loftus & Burns, 1982; Strange et al., 2003) and ‘high priority events’ (e.g., names of famous people, typically with an explicit instruction to attend to these words; Saufley & Winograd, 1970; Schulz, 1971; Tulving, 1969) retroactively impair memory. Retroactive impairment by aversive stimuli has been linked to release of noradrenaline (Hurlemann et al., 2005; Hurlemann et al., 2007), which is also released by salient/unexpected stimuli irrespective of valence (Berridge & Waterhouse, 2003; Kafkas & Montaldi, 2018; Yu & Dayan, 2005). Taken together, this suggests surprise will impair memory for surrounding episodic elements.

However, there are reasons to think the opposite: that arousing stimuli can retroactively enhance rather than impair memory. This is supported by studies revealing retroactive memory enhancement driven by reward (Braun et al., 2018; Murayama & Kitagami, 2014; Patil et al., 2017) and novelty (Ballarini et al., 2009, 2013; Fenker et al., 2008; Wang et al., 2010). In rodents, the retroactive effect of novelty is mediated by dopaminergic modulation of the hippocampus (Redondo & Morris, 2011; Wang et al., 2010; Yamasaki & Takeuchi, 2017). Surprising occurrences, like rewarding or novel ones, lead to dopaminergic activity (Barto et al., 2013; Horvitz, 2000; Ungless, 2004), suggesting surprise may also retroactively enhance memory. Moreover, a recent preprint found that episodic prediction error (a mismatch with a previous presentation of the stimulus) leads to retroactive memory enhancement (Sinclair et al., 2021). Other studies have found that aversive stimuli too may retroactively enhance, rather than impair, memory (Anderson et al., 2006; Dunsmoor et al., 2018; Dunsmoor et al., 2015; Smith & Beversdorf, 2008). Together this leads to the opposite prediction, that surprise will enhance memory for the surrounding episodic elements.

According to Event Segmentation Theory (Zacks et al., 2007), people segment continuous experience into distinct events, separated by event boundaries, which serve as an organising principle of long-term memory (Kurby & Zacks, 2008). If events are encoded as cohesive units, an arousing or surprising occurrence may retroactively affect memory for all preceding elements within the same event, but have no effect on memory for the preceding event. For example, a recent study found that fear conditioning enhanced memory for objects in the conditioned category, but only if they were presented within the same encoding block (Dunsmoor et al., 2018). Going back to the restaurant example, perhaps you would remember the starter well, but memory for your route to the restaurant would be unaffected or impaired.

To test potential retroactive effects of surprise in naturalistic experience, we designed a bespoke stimulus set, comprising stop-motion films (sequences of still photographs) depicting everyday actions, and clearly divided into distinct scenes. Some of the scenes had two versions—one with a surprising action, such as brushing teeth with rhubarb, and one with a neutral one, such as brushing teeth with a toothbrush. Because these scenes were identical aside from the target action, we were able to test retroactive effects of surprise in a naturalistic yet highly controlled manner, comparing memory for preceding actions within the same event or a preceding event. This enabled us to test whether surprise retroactively enhances, impairs, or does not affect memory, and whether the extent of the effect is limited to actions within the same event. While the main focus was on retroactive effects, the experimental design enabled us to test for proactive effects as well.

Another potential boundary condition concerns the delay between encoding and retrieval of memories. Many studies that found retroactive effects of salient stimuli only observed those effects when memory was tested after 24 hours; not immediately after encoding (Braun et al., 2018; Dunsmoor et al., 2018; Dunsmoor et al., 2015; Murayama & Kitagami, 2014; Patil et al., 2017). This suggests that salient stimuli affect the post-encoding consolidation of preceding items. We therefore tested memory both immediately following encoding and after a 24-hour delay (in separate experimental groups) to assess whether any observed effect is consolidation-dependent.

Thus, the primary aims of the study were to address the question of whether surprise affects memory for the entire event in which it occurred, and whether this effect is beneficial or detrimental. A secondary question concerned the basic unit of encoding. For example, if a surprising occurrence enhances memory for the entire event, but not preceding events, it would suggest the event is registered to memory as a cohesive unit. In contrast, if memory for the surprising element is enhanced but memory for preceding elements is unaffected or impaired, it would suggest each episodic element is registered to memory separately, perhaps bound together only through a shared context (Dubrow et al., 2017; Howard & Kahana, 2002; Polyn et al., 2009). Finally, if surprise produces a new event boundary, we may find, for example, enhanced memory for the surprising and preceding elements, but unaffected or even impaired memory for following elements (which would have been parsed into a new event).

## Method

### Participants

Participants were recruited through the online research platform Prolific (https://prolific.ac/), using the following eligibility criteria: ages 20–40 years; UK nationality; minimum of secondary school qualification; normal or corrected-to-normal vision; English as a first language; no diagnosis of mild cognitive impairment, dementia, or mental illness. UK nationality was added as a criterion as some elements of the films (e.g., use of Marmite) may be culture-specific. The experiment was approved by the Cambridge Psychological Research Ethics Committee and by the Cognition and Brain Science Unit’s Web-Based Experiment Management Committee. Informed consent was obtained from all participants prior to their participation.

### Sample size determination

Sample size was determined using the Bayesian “sequential design with maximal *n*” approach suggested by Schönbrodt and Wagenmakers (2018). According to this approach, data are collected until reaching either (a) the desired level of evidence or (b) the predefined maximal number of participants. The level of evidence is defined as the Bayes factor (BF) in favour of the alternative or in favour of the null (the higher of the two), with a BF of 3–10 typically considered moderate evidence and a BF of >10 considered strong (Lee & Wagenmakers, 2013). The experiment was run in batches of 24 participants per group. After each batch, we calculated the BF, relative to the null hypothesis, for effects of interest involving surprise in the primary analysis (henceforth ‘ANOVA testing for retroactive effects of surprise’). These include: a main effect of retroactive modulation by surprise; an interaction of action-type and surprise; and a three-way interaction of surprise, action-type, and delay (action-type referring to preceding actions in the same vs. a previous event and delay referring to immediate testing vs. 24-hr after study). The stopping criterion was defined as reaching a BF of 6 in favour of the alternative (BF10) for one of these tests, or reaching 6 in favour of the null (BF01) in all tests, or when the predefined maximal number of participants (*n* = 384: 8 batches of 24 per group) was reached (due to feasibility limits). The stopping criterion was reached at the predefined maximal number of batches, such that the final number of participants prior to exclusion was 384 (192 in each group).

### Stimuli

**Fig. 1:**
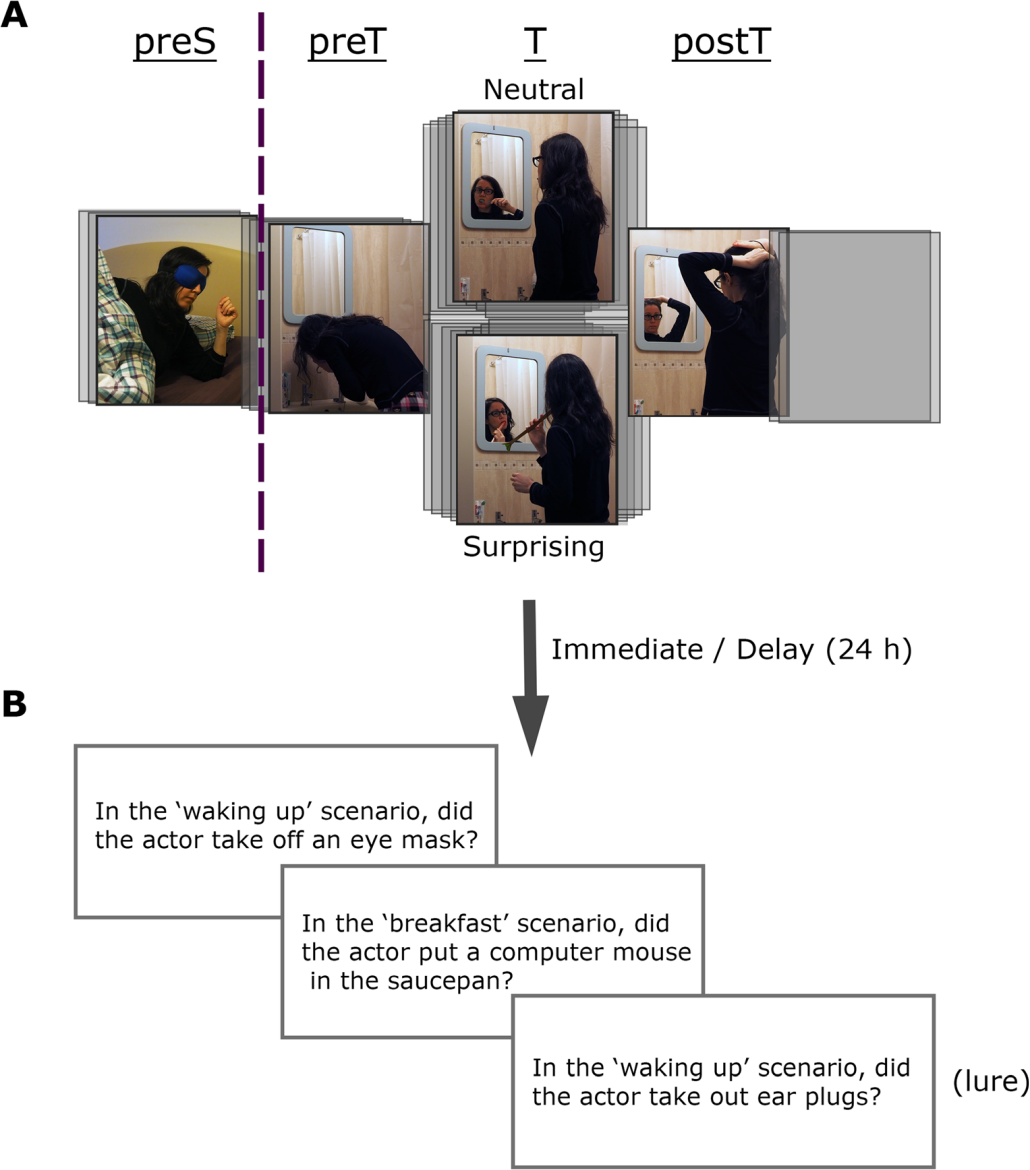
Experimental paradigm. **a** An example of a pair of scenes (pre-scene + target) presented in the study phase. Each target scene had two versions that were identical, aside from the target action, which could be either neutral (brushing teeth with a toothbrush) or surprising (brushing teeth with rhubarb). **b** Example of questions in the test phase (which took place either immediately or 24 hr after study). The test included one question about a pre-scene action (preS) and three questions about target scene actions (preT, T, postT). Each of the four test questions had a corresponding lure question. Participants answered on a confidence scale from −2 (*confident it did not occur*) to 2 (*confident it occurred*)

Participants were presented with three stop-motion films (photo series presented at 6 frames per second), each composed of scenes from the life of a different actor (see Fig. 1a). The films were designed to be very distinct from one another: they were filmed with different actors, in different environments and depict different scenarios and actions. Each scene depicted a series of daily actions, with a subset of the scenes (target scenes) having two versions—one with a surprising action (e.g., brushing teeth with rhubarb) and one with an equivalent neutral one (brushing teeth with a toothbrush). Importantly, the rest of the scene (before and after the target action) was identical in the two versions. Each target scene was preceded by a semantically related non-surprising scene (pre-scene), which had a single version. In addition, a few filler scenes (single-version, neutral) were dispersed in the longer movies, to slightly break up the scene-pair pattern. Film 1 had three target scenes (duration 30.8–39.3 s, mean 35.2 s) and three pre-scenes (duration 12.2–18.3 s, mean 15.8 s); Film 2 had eight target scenes (duration 11.7–27.2 s, mean 19.2 s), eight pre-scenes (duration 10.3–21.5 s, mean 15.1 s), and two filler scenes (duration 5.5, 10.2 s); Film 3 had seven target scenes (duration 26.3–41.5 s, mean 31.8 s), seven pre-scenes (duration 11.5–36.3 s, mean 19.5 s), and two filler scenes (duration 7.7, 10 s). The total duration of the films was Film 1: 2 m 33 s, Film 2: 4 m 50 s, Film 3: 6 m 16 s. As each target scene had two versions, each film could have multiple versions, depending on the set of target scenes presented in their surprising version and the set of target scenes presented in the neutral version. For example, as Film 1 has three target scenes, it has eight (2^3^) possible versions. Four versions of each film were used, chosen with roughly half surprise targets and half neutral targets. The four versions consisted of two pairs, such that for each version its mirror version was also used (the division to surprise/neutral was reversed). Moreover, the versions were created such that surprising and neutral target scenes were preceded by the same numbers of surprising/neutral targets (thus any carryover effects from the preceding target did not differ between conditions of interest). The four versions were counterbalanced across participants. The order of the films was also counterbalanced (leading to 24 variants of each version and order). However, due to a combination of participants stopping the experiment in the middle and the exclusion criteria, the final set of participants was not fully counterbalanced. After applying the exclusion criteria, the number of participants who viewed the films in each of the 48 version × order × delay combinations ranged from 4 to 10. When collapsing over order, the number in each version × delay combination ranged from 39 to 50; when collapsing over version, the number in each order × delay combination ranged from 22 to 32.

### Experimental design

The experiment was written in JavaScript, using the jsPsych library (de Leeuw, 2015; https://www.jspsych.org/) and set up using JATOS (Lange et al., 2015; http://www.jatos.org/).

Participants were presented with all three films (study phase; see Fig. 1a) followed by Yes/No questions (test phase; see Fig. 1b) and an assessment of surprise (surprise-assessment phase). For the delay group, the test phase (and surprise assessment) was on the following day (20–28 hours after study) instead of immediately following the study phase. Following each film (during the study phase), participants were presented with validation questions about the preceding film (in random order). Validation questions included one simple question per target scene (e.g., ‘was there a car-washing scene?’) and one lure (‘was there a tire-changing scene?’), aimed at verifying that participants had attended to each part of the films. Additionally, during the test phase, 10 extremely easy general-knowledge catch questions (‘Did it rain sometime during the last five years?’) were interspersed among the test questions to verify participants were not answering randomly at test. Test questions (presented in the test phase) probed specific actions that occurred within the scenes (see Fig. 1b)—one question for each pre-scene [preS] and three questions for each target scene (about a pre-target action [preT], the target action itself [T] and a post-target action [postT]). One of the questions of interest was whether the effects of surprise differ between preS and preT actions. However, preS questions are inherently more distant from the target (*M* = 17.1s, *SD* = 6.4s vs. *M* = 8.3s, *SD* = 4.4 s), which could account for any potential differences. The paradigm was designed with this issue in mind, ensuring there was large overlap between the question types (15/36 questions in an overlapping range) to enable accounting for effects of temporal distance. As each target scene could be presented in its surprising version or its neutral one, each question type could be further divided by surprise—preS-S, preT-S, T-S, postT-S when the surprising target was presented, and preS-N, preT-N, T-N, postT-N when the neutral target was presented. Each test question had a parallel lure question about an action that could have occurred at the same time in the scene, and did not occur in any of the films. During test, the questions about each film were presented separately, by order of film presentation, following a screen indicating which film was being probed (with a picture of the actor). Question order within each film was random. Each question described an action, and participants were requested to indicate whether it occurred on a scale of −2 to 2 (confident it did not occur/think it did not occur/do not remember/think it occurred/confident it occurred). Two measures of memory were used in each of the analyses described below: (1) a measure of Pr, number of hits (studied actions given a score of 1/2) minus number of false alarms (lure actions given a score 1/2), and (2) a measure of high-confidence Pr (difference between high-confidence hits and high-confidence lures) as a secondary analysis. During the surprise-assessment phase, participants were presented with a description of each target action (including both surprising and neutral ones), accompanied by a still frame depicting that action. For each action they were asked to indicate the degree of surprise (0/1/2/3 = did not notice the action/unsurprising/somewhat surprising/very surprising).

Prior to watching the first film, participants were instructed that they would be questioned about the actors’ actions, and they were presented with a brief demonstration including a pair of scenes (with no surprise) and four sample questions. They were not informed beforehand that there would be surprising actions, in order to maximise the degree of surprise.

### Exclusion criteria

Participants’ performance on the validation questions and catch questions was used to assess their attentiveness. Only participants with a Pr of at least 0.75 on the validation questions and at least 8/10 correct catch questions were included in the analysis. The validation questions were designed to be extremely easy to answer if the participant paid attention to the films (even for a participant with poor memory). As we used internet testing, we had less control over the participants’ surroundings, and extra measures were taken to ensure participants were indeed attending the films (and not engaged in another activity). Using these criteria, 44 participants were excluded from analysis—20 of the immediate group and 24 of the delay group.

### Ceiling/floor performance

After the first batch of participants (*n* = 24 per group), we assessed performance in order to determine whether adaptations should be made to the design. Performance was assessed after applying the exclusion criteria (removing two participants from the immediate group and three participants from the delay group). Floor performance was defined as moderate evidence (BF >3, one-tailed comparison) in favour of the null when testing for a difference between the number of hits and false alarms (Pr = 0). The strategy set in place in case of floor performance for the non-target questions (preS, preT, and postT) in the first batch of the delay group was to focus the experiment on the immediate group. If performance had been at chance in the immediate group as well, we planned to alter the design to have three study–test cycles, with memory for each film being probed immediately following that film. In addition, we verified that surprising target actions are indeed recalled better than neutral ones (T-S > T-N). In case of moderate evidence in favour of the null (BF >3, one-tailed comparison, collapsing across groups), scenes with the smallest difference in performance would have been replaced with new scenes, with a more salient surprising target action. Ceiling performance was defined as Pr >0.9 for non-target questions in at least 75% of participants. If the first batch of participants had reached the ceiling performance criterion (in either group), we planned to add scenes to the films to make the experiment harder. No changes were required after applying these criteria to the first batch of participants, so it was included in analysis. There was no evidence of floor performance in either group (8×10^-10^ in the immediate group, 3.6×10^-11^ in the delay group) and no participants exhibiting ceiling performance. There was also no evidence in favour of the null when comparing memory for surprising target actions to memory for neutral ones (BF01 = 0.0001).

### Identifying event boundaries

The films are separated into distinct scenes, which are likely to be perceived by participants as distinct events, given that they are accompanied by a change in location (Zacks et al., 2009). To verify this, a separate group of 18 participants viewed the films and indicated with a button press when they experienced an event boundary. This was run during Stage 1, as an a priori verification of boundaries. The specific instruction was ‘press SPACE whenever, in your experience, one event (a narrative unit) ends and a new one begins. Try to press SPACE as soon as possible after you notice a transition to a new event.’ Before viewing the films, participants were presented with a screen that changed colour and asked to press a button when the colour changes. This was used to calculate the per-participant response time, as response times may be affected by issues such as system or connection speed that differ between participants. The event boundary button presses were then corrected using the estimated response time. Each of the scene changes was identified as a boundary by a minimum of 11/18 participants while the maximal agreement at any other time-point was of 4/18 participants (within a 3-s sliding window). Further details about the event boundary identification can be found in the Supplementary Analysis.

### Statistical analyses

Bayesian statistics were used to assess evidence in favour of the alternative versus the null. For each statistical test, BF10 was computed, which estimates how likely the alternative is relative to the null, given the observed data. Similarly, BF01 (equivalent to 1/BF10) indicates how likely the null is compared with the alternative. All participant-averaged statistics were calculated using the BayesFactor package (Morey et al., 2018; ttestBF and anovaBF, https://cran.r-project.org/web/packages/BayesFactor/index.html), using the default scale parameter of √2/2 for Bayes factor calculations in R (R Core Team, 2016). Each of the analyses was run once using the regular Pr (all hits − all false alarms) and once using the high-confidence Pr (high confidence hits − high confidence false alarms). For each hypothesis, evidence was assessed both in favour of the null (B01) and in favour of the alternative (B10). Single-trial analyses were run using the brms package (Bürkner, 2017; https://cran.r-project.org/web/packages/brms/index.html), with a Bernoulli family (i.e., distribution of the dependent variable) and the default logit link function. Generic, weakly informative priors were used [student_t(1,0,10) for the intercept and student_t(5,0,2.5) for the coefficients]. Hypothesis testing was then conducted using the bayes_factor function of the brms package, comparing models with/without each effect of interest.

#### Retroactive and proactive effects of surprise

The primary question of interest was whether surprise retroactively (or proactively) affects memory, and if so, which factors modulate the effect. Two Bayesian three-way ANOVAs (anovaBF) were used to test the effect of question type and surprise on memory performance (Pr). The first ANOVA tested whether surprise differentially affects preceding actions within versus across events—a 2 × 2 × 2 ANOVA with Pr as the dependent measure, a within-participant factor of surprise, a within-participant factor of within/between event (preT vs. preS), and a between-participant factor of delay (immediate vs. delay). The second ANOVA tested whether surprise differentially affects preceding actions versus following ones (same as first ANOVA, but comparing preT with postT). The ANOVAs were followed by secondary analyses separately assessing the effect within each question type (preS, preT, postT). These consisted of a *t* test (ttestBF, two-sided hypothesis) for the effect of surprise (Pr Surprise − Pr Neutral) within each group, a *t* test collapsing across groups and a *t* test comparing the two groups.

#### Potential factors modulating retroactive/proactive effects

If surprise exerts a retroactive (or proactive) effect on memory, there are several factors that may determine the strength of this effect. According to the proposed method, if the primary analysis revealed evidence that surprise had a retroactive/proactive effect, the following factors would be added as predictors in a secondary analysis:

1. The degree of surprise (estimated in the surprise assessment phase).
2. The time between the action and the nearest preceding surprise. This accounts for temporally graded carryover effects from preceding scenes.
3. The time between the action and the nearest subsequent surprise. This factor controls for differences between probed actions in their proximity to the following surprise.
4. The expectancy of surprise. Because ‘surprisal’ may diminish over time, earlier surprising occurrences may have a stronger effect (Hirshman, 1988; Hirshman et al., 1989; Reggev et al., 2018). This can be accounted for by adding a predictor of the number of preceding surprising occurrences (across films).

However, as the primary analysis did not reveal a clear retroactive or proactive effect, the above analysis was not run.

### Post hoc control analysis

#### Proactive effects of surprise—Single-trial analysis

The single-trial analysis was added as a follow-up in Stage 2, after data collection. After finding strong evidence of a proactive effect, we ran an additional analysis to test whether this could have been due to counterbalancing issues. Due to participants stopping the experiment in the middle, in addition to exclusion criteria, the final set was not fully counterbalanced in terms of order (the presentation order of the films) and version (which specific set of scenes were presented in their surprising version). Thus, the proactive effect could have arisen by chance due to item effects (e.g., a difference in the memorability of different actions). To account for this, we ran a single-trial analysis, in which both participant and action were included as random effects. The model included a binary dependent variable ansOld (indicating whether the participant recognised the action as old); an interaction of isFoil (whether the question was a foil) with the action presentation order, surprise (whether the scene-pair contained a surprising occurrence), action-type (preT/postT), and group; two random effects of participant and action. Note that the inclusion of the isFoil fixed effect, specifically its interaction with the other factors, adjusts for false alarm rate in a manner comparable with the use of Pr in the previous analyses. To test evidence in favour of each effect, we compared a model that included an interaction of isFoil with that factor with a model that did not have the interaction. The full model was:

$$ \mathrm{ansOld}\sim \mathrm{isFoil}\ast \left(\mathrm{surprise}\ast \mathrm{action}-\mathrm{type}\ast \mathrm{group}+\mathrm{action}-\mathrm{order}\right)+\left(1|\mathrm{subj}\right)+\left(1|\mathrm{action}\right), $$

with a logistic linkage function. This analysis required the brms package (Bürkner, 2017; https://cran.r-project.org/web/packages/brms/index.html), as opposed to the planned analyses which use the BayesFactor package (Morey et al., 2018; ttestBF and anovaBF, https://cran.r-project.org/web/packages/BayesFactor/index.html).

## Results

Using the “Bayesian sequential design with maximal *n*” approach (Schönbrodt & Wagenmakers, 2018), data collection was stopped at the predefined maximal number of batches (i.e., “maximal *n*”), such that the final number of participants was 340 (172 in the immediate group and 168 in the delay group), after excluding 44 participants who met our a priori exclusion criteria (see Methods).

### Memory performance

For surprising target actions, the mean overall accuracy (Pr = hit-rate − false alarm rate) was 0.75 (*SEM* = 0.02) / 0.71 (*SEM* = 0.02) for the immediate/delay group, and for neutral targets, Pr was 0.51 (*SEM* = 0.02) / 0.39 (*SEM* = 0.02) respectively. For non-target actions (preS, preT, postT), Pr was 0.52 (*SEM* = 0.007) / 0.4 (*SEM* = 0.007). The full set of accuracy results (with memory performance for each action type, further divided by surprise, as well as the results of the high-confidence analysis) is provided in Table 1. The full set of hit rates are in Supplementary Table 2.

**Table 1:** Accuracy (Pr) in each condition

		Pr		high-confidence Pr	
		Immediate	Delay	Immediate	Delay
preS	N	*M* = 0.53, *SEM* = 0.02	*M* = 0.42, *SEM* = 0.02	*M* = 0.47, *SEM* = 0.02	*M* = 0.36, *SEM* = 0.01
	S	*M* = 0.53, *SEM* = 0.02	*M* = 0.42, *SEM* = 0.02	*M* = 0.47, *SEM* = 0.02	*M* = 0.34, *SEM* = 0.02
preT	N	*M* = 0.52, *SEM* = 0.02	*M* = 0.41, *SEM* = 0.02	*M* = 0.48, *SEM* = 0.02	*M* = 0.34, *SEM* = 0.02
	S	*M* = 0.54, *SEM* = 0.02	*M* = 0.41, *SEM* = 0.02	*M* = 0.48, *SEM* = 0.02	*M* = 0.32, *SEM* = 0.02
T	N	*M* = 0.51, *SEM* = 0.02	*M* = 0.39, *SEM* = 0.02	*M* = 0.43, *SEM* = 0.02	*M* = 0.31, *SEM* = 0.02
	S	*M* = 0.75, *SEM* = 0.02	*M* = 0.71, *SEM* = 0.02	*M* = 0.72, *SEM* = 0.02	*M* = 0.68, *SEM* = 0.02
postT	N	*M* = 0.52, *SEM* = 0.02	*M* = 0.40, *SEM* = 0.02	*M* = 0.44, *SEM* = 0.02	*M* = 0.33, *SEM* = 0.02
	S	*M* = 0.48, *SEM* = 0.02	*M* = 0.33, *SEM* = 0.02	*M* = 0.39, *SEM* = 0.02	*M* = 0.27, *SEM* = 0.01

*Note.* The mean Pr and high-confidence Pr for each condition – action-type × surprise × group.

**Fig. 2:**
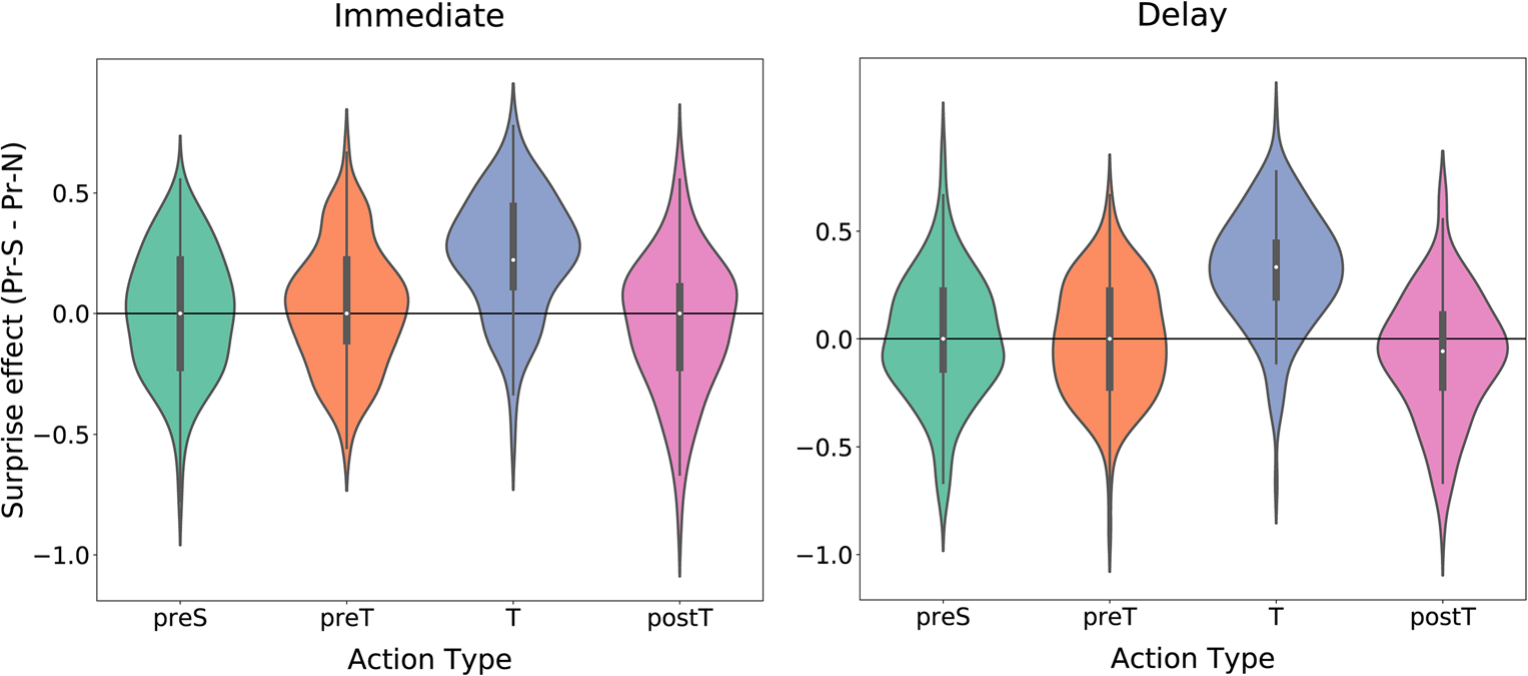
Surprise effect on memory for diferent action types. The effect of a surprising target on memory accuracy (the difference between Pr when the scene-pair included a surprising target and Pr when it included a neutral one), divided by action-type (preS/preT/T/postT), and group (Immediate/Delay)

Most importantly, the effect of surprise on accuracy (defined as Pr when the target was neutral subtracted from Pr when the target was surprising) was very small for non-target actions (see Fig. 2)—that is, there was little suggestion of effects of surprise on memory for surrounding actions. We test these effects below. In contrast, there was overwhelmingly strong evidence that surprising targets are remembered better than neutral ones (BF10 = 2.2×10^24^ in the immediate group and BF10 = 2.4×10^31^ in the delay group), indicating the lack of an effect on non-target actions was not due a general inability to identify surprise effects in the experimental design.

#### No retroactive effects of surprise

Contrary to our hypothesis, we found evidence that surprising occurrences do not modulate memory for preceding actions, even within the same event. We ran a Bayesian ANOVA testing whether memory for actions was modulated by a surprising occurrence later in the same scene-pair, including both preS actions (in the scene preceding the surprise) and preT actions (a preceding action within the same scene). The ANOVA included three factors—a within-subject factor of surprise (whether or not the target action was surprising), a within-subject factor of action type (preS/preT), and a between-subject factor of delay (memory test immediately after study or 24 hours later). The stopping criterion for data collection was finding evidence (BF ≥6) in favour of one of three tests—a main effect of surprise, a surprise × action-type interaction, or a three-way interaction—or evidence in favour of the null in all three tests. After collecting eight batches of participants, we reached the stopping criterion, with evidence in favour of the null for all tests (BF01 = 10 against a main effect of surprise; BF01 = 9.6 against a surprise × action-type interaction [see Supplementary Fig. 4]; BF01 = 6.9 against a three-way interaction). The high-confidence analysis (treating only high-confidence responses as hits/false alarms) yielded similar results, with evidence in favour of the null in all tests (BF01 = 6.7 against a main effect of surprise; BF01 = 10.5 against a surprise × action-type interaction; BF01 = 7.7 against a three-way interaction).

#### Inconclusive evidence of proactive interference

To assess whether surprise proactively affects memory, we ran a similar three-way ANOVA, here comparing preT actions with postT actions (following actions in the same scene as the target). There was inconclusive evidence for a main effect of surprise (BF10 = 1.3), strong evidence for a surprise × action-type interaction (BF10 = 26.2; see Supplementary Fig. 4), and moderate evidence against a three-way interaction (BF01 = 7.9). Follow-up tests showed that this was due to lower memory for postT actions that followed a surprising target, when collapsing across groups, and no effect for preT actions (BF10 = 59.1 for postT and BF01 = 12.4 against preT). While there was no evidence of a difference in the postT surprise effect between groups (BF01 = 4.6), this still seemed to be driven primarily by the delay group, as there was strong evidence for a surprise effect in postT questions in the delay group (BF10 = 20) and inconclusive evidence against an effect in the immediate group (BF10 = 0.5).

We ran a similar analysis treating only high-confidence responses as hits/false alarms. The ANOVA yielded different results, with strong evidence in favour of a main effect of surprise (BF10 = 52.5), weak evidence in favour of a surprise × action-type interaction (BF10 = 3.1) and strong evidence against a three-way interaction (BF01 = 16). However, follow-up *t* tests revealed a similar pattern as the regular analysis, with lower memory for postT actions that followed a surprising target, when collapsing across groups, and no effect for preT actions (BF10 = 203 for postT and BF01 = 11.4 against preT). There was again no evidence of a difference in the postT surprise effect between groups (BF01 = 6.7), but the effect seemed to be driven primarily by the delay group, with strong evidence for a surprise effect in postT questions in the delay group (BF10 = 15.4) and inconclusive evidence for an effect in the immediate group (BF10 = 1.6).

While these results seemed to be indicative of a proactive effect of surprise, at least when memory was tested after a delay, we probed these results further. This additional analysis was not part of the Stage 1 specification, and was added since the participant pool was not fully counterbalanced (due to participant dropout during the experiment and exclusion of participants based on the predefined criteria). For each combination of group × film order × film version there were 4–10 participants after applying the exclusion criteria. To assess the reliability of the results, we ran a single-trial analysis that accounted for any effects of order/version. In this analysis, there was evidence against a main effect of surprise (BF01 = 6.4) and against a three-way interaction (BF01 = 10), and most importantly—only anecdotal evidence in favour of a surprise × action-type interaction (BF10 = 2.7). In the equivalent high-confidence analysis there was moderate evidence for a main effect of surprise (BF10 = 4.7), evidence against a surprise × action-type interaction (BF01 = 9.1) and evidence against a three-way interaction (BF01 = 6.5). While the discrepancy between the single-trial analysis and participant-averaged analysis could have arisen from differences in choice of priors, given the inconclusive results of the single-trial analysis we cannot conclude there is a reliable proactive effect.

## Discussion

In this study, we tested whether memory for the episodic elements of naturalistic films can be modulated independently—in this case, by the presence of a surprising event—or whether the fate of elements within the same event is tied together. Using bespoke stop-motion films, in which a single element could be replaced by a surprising one, we found clear evidence that surprising elements are better remembered, but evidence against the hypothesis that this spreads to preceding elements, either within the same event or in a preceding event. The same pattern was observed whether memory was tested immediately after study or when it was tested 24 hours later. There was a suggestion that memory for elements following the surprise is impaired, particularly after a delay, but evidence of this was inconclusive.

The lack of a retroactive effect seems at odds with previous studies that identified either impairment or enhancement when using arousing stimuli, including aversive stimuli (Bornstein et al., 1998; Hurlemann et al., 2005; Knight & Mather, 2009; Loftus & Burns, 1982; Strange et al., 2003), rewarding stimuli (Braun et al., 2018; Murayama & Kitagami, 2014; Patil et al., 2017), and oddballs (‘high-priority events’; Saufley & Winograd, 1970; Schulz, 1971; Tulving, 1969). Since most of these studies entailed incidental encoding, rather than the intentional encoding used in the current study (which was necessary to achieve reasonable memory performance), one possibility is that different encoding strategies explain the different results. However, another possibility is that the mechanisms by which these types of stimuli affect memory differ from those of surprise. One study, more similar to our own, tested effects of incongruent components in an event (sequence of pairwise associations) on memory for other elements (Frank et al., 2018). While they found enhanced memory for associations between elements in the incongruent condition, this was driven by reduced interference from the incongruent element. In line with our results, when testing (incidental) item recognition they found no effect of an incongruent element on memory for the other elements in the event.

The primary goal of the study was to determine whether enhanced memory for an element within an event would spread to the entire event. If we had found evidence the effect spreads to surrounding elements within the same event (but not beyond), it would have supported the hypothesis that elements are bound together and registered to memory as a cohesive unit. This hypothesis is based on findings of hippocampal activity at event boundaries, often attributed to the binding together of the preceding event (Baldassano et al., 2017; Ben-Yakov & Dudai, 2011; Ben-Yakov et al., 2013; Ben-Yakov & Henson, 2018; Cooper & Ritchey, 2020; DuBrow & Davachi, 2016; Kurby & Zacks, 2008; Lu et al., 2019), as well as studies identifying reactivation of encoding patterns at the offset of events (Silva et al., 2019; Sols et al., 2017). Moreover, studies of episodic retrieval have found that events are retrieved and forgotten holistically (Horner & Burgess, 2013, 2014; Joensen et al., 2019; Jonker et al., 2018), reinforcing the idea of holistic encoding. Here however, the evidence against retroactive enhancement supports the alternative hypothesis—namely, that each element is encoded independently, and that the elements are bound together only through a shared context (Bladon et al., 2019; Howard et al., 2005; Polyn et al., 2009; but see Dubrow et al., 2017, for a suggested reconciliation of the two accounts). One possibility is that the hippocampal activity observed at event boundaries does not reflect binding; another is the surprise-driven strengthening of memory occurs online (during the event, when the surprising element occurs), independently of a binding process that occurs at the end of the event.

A third possible explanation for the lack of retroactive enhancement is that surprise itself creates an event boundary (Antony et al., 2021), sectioning off the preceding elements as a distinct event. To explore this possibility, we tested whether surprise reduces the dependency between elements that precede/follow it (as a preliminary exploratory analysis, presented in the Supplementary Analyses). Previous studies have found retrieval dependency between episodic elements in the same event, such that if one element in an event is retrieved, the others are more likely to be retrieved as well (Horner & Burgess, 2013, 2014). We found a similar effect in neutral events, with strong dependency between pre-target and post-target actions. This dependency was significantly reduced (although remained significant) when these actions straddled a surprising action. As a control, we tested the effect of surprise on dependency between pre-target and pre-scene actions (which did not straddle the target action), and did not observe a significant reduction. While the pre-target/post-target effect was not significantly stronger than the pre-target/pre-scene, these results are in line with the possibility that surprise acts as a boundary. We also tested a further sample of participants to see whether people subjectively experience surprising moments as event boundaries. Using standard instructions for marking the presence of a boundary, we found no evidence that people did experience surprising actions as a boundary. This discrepancy could arise because the instructions given for online, subjective demarcation of boundaries do not precisely match the factors that actually cause event segmentation in memory. Thus while the exploratory dependency analysis for participants’ memory lends some support for the hypothesis that surprise can segment events in memory, further research is required to draw more definitive conclusions, for example by probing binding between episodic elements with tests of cued recall/recognition and temporal order memory (e.g., DuBrow & Davachi, 2014; Heusser et al., 2018; Horner et al., 2016; Rouhani et al., 2020; Sols et al., 2017; Swallow et al., 2009).

In summary, across a large number of participants who watched naturalistic films, we find conclusive evidence that a surprising element does not modulate memory for preceding elements within the same event, suggesting that it is possible to independently manipulate memory for a single element within an event without affecting the others. This suggests episodic elements are encoded to memory separately, each as it is encountered, rather than all together as a cohesive unit at the end of the event. However, there are two additional accounts that merit further investigation: (1) that the enhancement afforded by surprise occurs during experience, but an additional and independent encoding process binds together events upon their conclusion, and (2) that surprise itself segments experience, acting as an event boundary in terms of its effects on memory.

## Supplementary Information

ESM 1 (PDF 662 kb)
