# Supplementary material for: The limited reach of surprise: Evidence against effects of surprise on memory for preceding elements of an event
Source: Psychon Bull Rev. 2021 Jun 25;29(3):1053–64. doi: 10.3758/s13423-021-01954-5 (PMC9166837; doi:10.3758/s13423-021-01954-5)
Supplement: Supplementary file 1 — (PDF 662 kb) [file 13423_2021_1954_MOESM1_ESM.pdf]

Supplementary Analyses

Exploratory Analyses

Methods

Dependency assessment

Following the results of the planned analysis, we ran an exploratory analysis to test whether surprising occurrences may constitute a boundary. To do so, we assessed dependency between actions in the same episode that straddled the target action, which could be surprising or neutral. Previous research has revealed increased dependency between the retrieval of elements of the same event (Horner & Burgess, 2013). Thus, we expect there to be lower dependency between preT and postT actions in the surprise condition relative to the neutral one if indeed surprise acts as a boundary, separating preT and postT actions into different events. To assess dependency between preT and postT actions, we fit generalized linear mixed-effects models (in a single-trial analysis) with the following formulas:

preT ~ postT + preS + T + group + (1|participant) + (1|scene)

postT ~ preT + preS + T + group + (1| participant) + (1|scene)

The data included an observation for each scene X participant, with four binary variables indicating hit/miss of each action in that scene (preS/preT/T/postT), and a binary variable of group indicating whether the participant belonged to the immediate/delay group. Participant and scene were random variables indicating the participant and scene of each trial. The models were fit using the glmer function (binomial family) of the lme4 package (Bates, Mächler, Bolker, & Walker, 2015, cran.r-project.org/package=lme4). The models were used to define two directional measures of dependency, postT->preT (the coefficient of the postT predictor when preT was the dependent variable) and preT->postT (the coefficient of the preT predictor when postT was the dependent variable). The bidirectional dependency was then defined as an average of the directional measures. Dependency between preT and preS actions, which served as a control, was calculated in the same manner.

The significance of dependency in each scene type (neutral/surprise) was calculated with permutation testing, comparing preT/postT dependency to the dependency calculated when randomly pairing preT actions with postT actions from a different scene (n=1000 iterations).

The effect of surprise on dependency was defined as a subtraction of surprise dependency from neutral dependency:

(preT/postT)N-(preT/postT)S

And significance was assessed in the same way, comparing the dependency-difference to random pairings.

The comparison between the effect of surprise on preT/postT dependency and preT/preS dependency (serving as a control) was defined as:

[(preT/postT)N-(preT/postT)S] – [(preT/preS)N-(preT/ preS)S]

And significance was assessed similarly.

Assessing surprise as a subjective boundary

The dependency analysis served to assess whether surprise acts as an event boundary in terms of its effect on subsequent memory performance. In a complementary analysis, we assessed whether surprise constitutes a boundary in terms of subjective experience. A group of 18 participants viewed the films with all target scenes presented in their surprising version and indicated with a button press when they experienced a boundary (using the same procedure as in the section 'Identifying event boundaries'). For each surprising action, we calculated the number of participants who identified a boundary between 500ms before the action (to account for slight variation in reaction time) and 3000ms after the action. We compared this to the average number of participants who identified scene changes as boundaries. For scene changes we used a smaller window (up to 2000ms following the scene change) as scene changes are better temporally defined. This analysis was run post-hoc, following the results observed for the planned analyses.

Results

Surprise reduces dependency between episodic elements

The finding that surprising elements do not modulate memory for other elements in the same event suggests that each element is encoded independently. However, an alternative possible interpretation is that surprise acts as an event boundary, segmenting the event such that preT and postT actions no longer belong to the same event as the surprising one. We explored this possibility, testing whether surprise decreases dependency between retrieval of preT and postT actions. A previous study identified dependency between retrieval of elements of the same event, such that there is an increased chance of remembering an element if other elements were remembered as well (Horner & Burgess, 2013). Based on this, we expect to observe dependency between retrieval of preT and postT actions. If surprise serves as a boundary, we would expect this dependency to be reduced when the event contains a surprising target. The analysis focused only on preT/postT dependency, and not the dependency of each with the target, as there was a very high hit-rate for surprising targets, with over half of the participants having at most one miss.

We first verified that there was a significant preT/postT dependency for neutral events, when comparing the dependency measure (see Methods) to the dependency when randomly pairing preT actions with postT actions from other events. This indeed revealed significant dependency ($p < 0.001$, Figure 3A). While for surprising events the dependency remained significant (Figure 3A), it was reduced relative to dependency in neutral events, and the difference was significant ($p < 0.001$, Figure 3B). As a control analysis, we tested the effect of surprise on dependency between preT and preS actions, where we do not expect to see an effect (since they do not straddle the surprise). Indeed the effect of surprise on preT/preS dependency did not reach significance ($p = 0.1$, Figure3C), although a

direct test of the difference in the surprise effect on preT/postT and preT/preS was not significant either (p = 0.33).

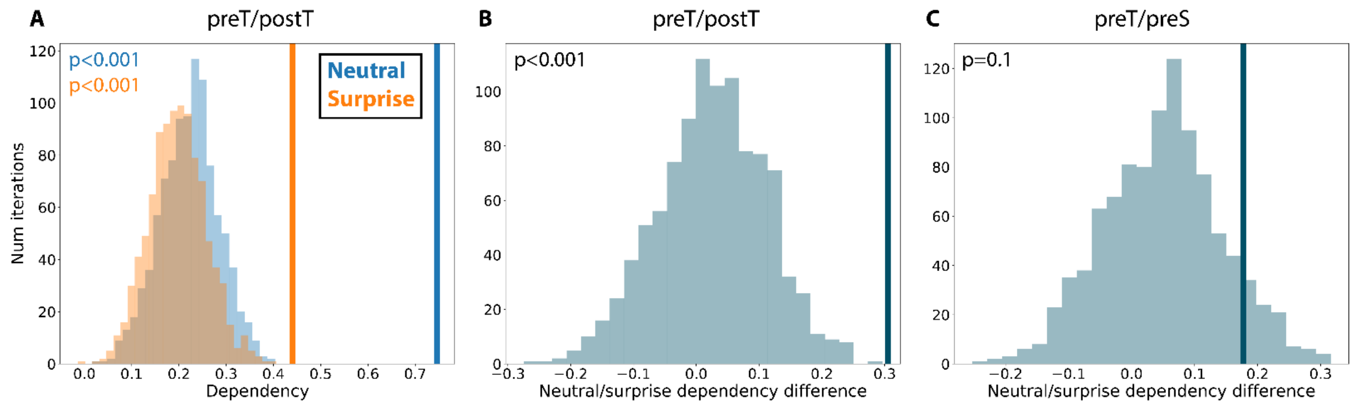

Supplementary Figure 1. Reduced dependency between episodic elements that straddle a surprising occurrence. (A) The preT/postT dependency in scenes with a neutral/surprising target (vertical line), compared to the dependency when randomly pairing preT actions with postT actions from other events. (B) The vertical line represents the difference in preT/postT dependency between scenes with neutral and surprising targets, compared to the dependency difference in random pairings. (C) Same as (B), looking at preT/preS dependency as a control.

Surprise is not experienced as a subjective event boundary

Following the results of the dependency analysis, which suggests surprise may act as an event boundary in terms of organisation of long-term memory, we asked whether surprise is perceived as a subjective event boundary. A group of 18 participants viewed the films with all targets presented in their surprising version and indicated when they experienced a boundary, with an instruction to indicate when one event/narrative unit ends and a new one begins. We did not find evidence that surprising actions are experienced as a boundary (Supplementary Figure 4). The average number of participants who identified a boundary at a surprising action was 0.9 (maximum of 3), similar to the number of participants who identified boundaries at the same time-points in the neutral version (M = 0.8, maximum again 3). In contrast, the average number of participants who identified boundaries at scene changes was 13.9, with a maximum of 18 (despite using a smaller window for the scene-change analysis).

Additional Supplementary Analyses

Event boundary identification

The films were composed of distinct scenes, which we presumed would be experienced by participants as distinct events. To verify this, a separate group of 18 participants viewed the films in their neutral version and indicated when they experienced a boundary. This served two purposes:

1. To verify that all scene changes were experienced as boundaries
2. To verify there were no additional boundaries, identified within scenes

For a qualitative appraisal, we first plotted the scene changes along with all button presses of all participants (corrected for reaction time). Consecutive button presses (separated by less than 2s) by the same participant were treated as a single button press, as participants sometimes accidentally pressed space for a long time, and this was recorded as multiple key presses.

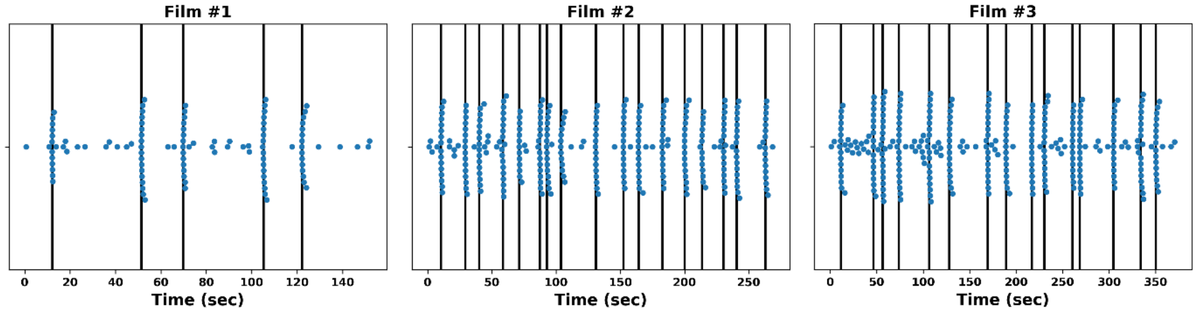

Supplementary Figure 2. Scene changes and subjective boundaries. Plots of the scene changes in each film (vertical black lines) along with all button presses of all participants (blue dots), corrected for each participant's reaction time.

These plots demonstrate that all scene changes, and no other points in time, were reliably identified by participants as boundaries. To quantify this, we counted the number of participants identifying a boundary in each time-point, using a sliding window (window size 3s, with a jump of 1s). The distribution of the number of participants identifying a boundary in each window (for all windows with at least one boundary) is plotted below:

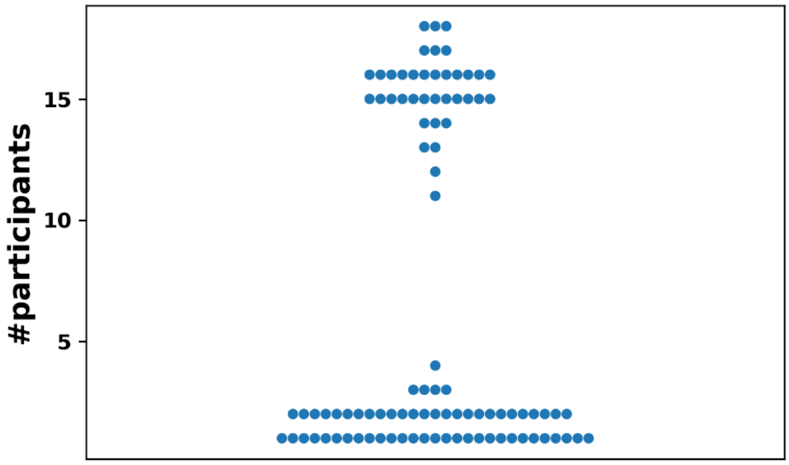

Supplementary Figure 3. Distribution of the number of participants identifying boundaries. Plot of all peak heights in the sliding window analysis. The peak height corresponds to the number of participants identifying a boundary in a given time-window. The top set of windows corresponds exactly to the set of scene changes.

When defining subjective boundaries using the sliding window analysis (defining boundaries as peaks identified by at least 10 subjects), the mean temporal distance between scene changes and the closest boundary was 491ms and the maximal distance was 1167ms.

Retroactive and proactive effects – plot

Complementary figure to Figure 2 of the main text, here showing the preT-preS and postT-preT differences in the surprise effect (equivalent to an interaction of surprise and action type in the 3-way ANOVAs).

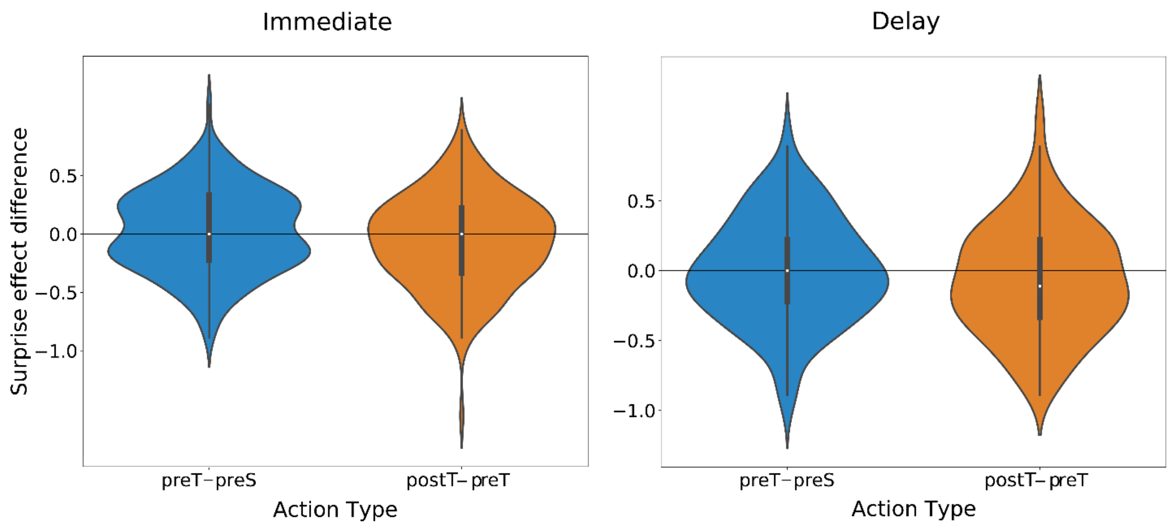

Supplementary Figure 4. Difference in surprise effect between action types. In blue, the difference between the surprise effect (Pr-S – Pr-N) on preT actions and the effect on preS actions. In brown, the difference between postT and preT actions. Both are presented for the Immediate group (left) and the Delay group (right).

Retroactive and proactive effects - follow-up tests

The full set of planned follow-up t-tests for the retroactive and proactive ANOVAs are presented in the table below.

	Immediate group	Delay group	Collapsed across groups	Comparison between groups
preS	0.09 / 11.7	0.09 /11.6	0.06 / 16.4	0.1 / 8.3
preT	0.2 / 5.6	0.09 / 11.5	0.08 / 12.4	0.2 / 5.5
postT	0.5 /1.9	20 / 0.05	59.1 / 0.02	0.2 / 4.6
preS (HC)	0.09 / 11.6	0.1 / 7.6	0.07 / 13.7	0.2 / 6.1
preT (HC)	0.09 / 11.2	0.1 / 7.8	0.09 / 11.4	0.1 / 7.7
postT (HC)	1.6 / 0.6	15.4 / 0.07	203 / 0.005	0.1 / 6.7

Supplementary Table 1. Planned follow-up t-tests for retroactive and proactive effects. The evidence for/against (BF10/BF01) a surprise-effect (Pr-S – Pr-N difference) in each action – separately for each group, collapsed across groups and a comparison between groups.

Memory performance – hit rates

The full set of hit rates, with a division both by action-type and by surprise.

	hit-rate		high-confidence hit-rate	
	Immediate	Delay	Immediate	Delay

preS	N	M = 0.66, SEM = 0.01	M = 0.61, SEM = 0.01	M = 0.52, SEM = 0.02	M = 0.43, SEM = 0.01
	S	M = 0.65, SEM = 0.01	M = 0.61, SEM = 0.02	M = 0.53, SEM = 0.02	M = 0.4, SEM = 0.02
preT	N	M = 0.66, SEM = 0.01	M = 0.62, SEM = 0.01	M = 0.53, SEM = 0.02	M = 0.39, SEM = 0.02
	S	M = 0.68, SEM = 0.01	M = 0.62, SEM = 0.02	M = 0.53, SEM = 0.02	M = 0.39, SEM = 0.02
T	N	M = 0.64, SEM = 0.01	M = 0.58, SEM = 0.01	M = 0.48, SEM = 0.02	M = 0.37, SEM = 0.02
	S	M = 0.79, SEM = 0.01	M = 0.78, SEM = 0.02	M = 0.74, SEM = 0.02	M = 0.71, SEM = 0.02
postT	N	M = 0.63, SEM = 0.01	M = 0.58, SEM = 0.01	M = 0.49, SEM = 0.02	M = 0.38, SEM = 0.01
	S	M = 0.59, SEM = 0.01	M = 0.53, SEM = 0.01	M = 0.45, SEM = 0.02	M = 0.33, SEM = 0.01

Supplementary Table 2. Hit-rate in each condition. The mean hit-rate and high-confidence hit-rate for each condition – action-type X surprise X group.

Assessing surprise as an event boundary

To test whether surprise is experienced as a subjective event boundary, we presented participants with the films in their surprising version, and asked them to indicate with a button press when they experienced a boundary. The same procedure was used as in the 'Event boundary identification' section of the Supplementary Analyses, with the exception that all target scenes were presented in their surprising version.

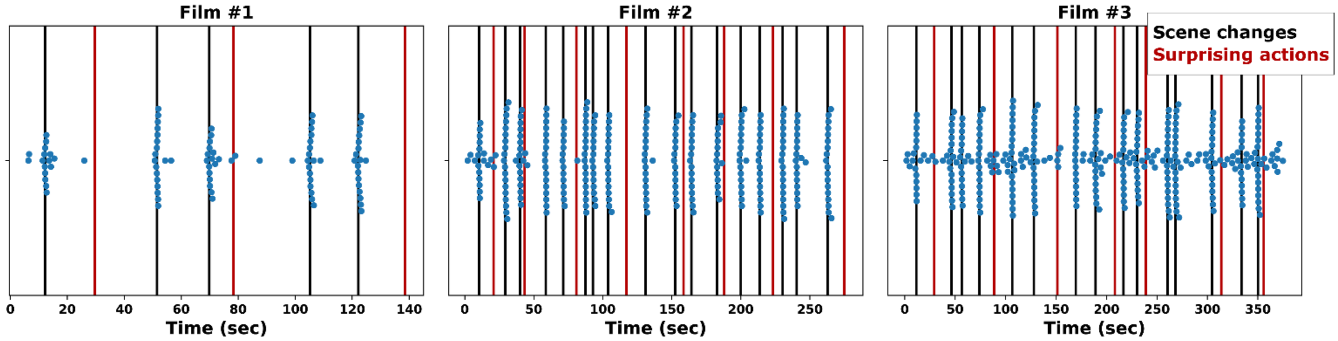

Supplementary Figure 5. Subjective markings of event boundaries in surprising version. The blue dots indicate points in time identified as event boundaries by observers who viewed all target scenes in their surprising versions. The vertical black lines indicate scene changes and the vertical red lines indicate a surprising action.
